# COVID-19 Coronavirus-Induced Atypical Pneumonia: Efficacy of the Monoclonal Antibody Bevacizumab in Moderate to Severe Cases

**DOI:** 10.7759/cureus.18317

**Published:** 2021-09-27

**Authors:** Greshaben Patel, David Pielykh, Sweetyben M Patel, Meet J Patel, Kaushal Bhavsar, Thoyaja Koritala

**Affiliations:** 1 Internal Medicine, B.J. Medical College, Ahmedabad, IND; 2 Internal Medicine, Odessa National Medical University, Odessa, UKR; 3 Internal Medicine, Interfaith Medical Center, Brooklyn, USA; 4 Pulmonary and Critical Care, Sola Civil Hospital, Ahmedabad, IND; 5 Internal Medicine, Mayo Clinic, Mankato, USA

**Keywords:** angiogenseis, bevacizumab, hypoxia, covid-19 pandemic, atypical pneumonia, acute respiratory distress syndrome

## Abstract

COVID-19 novel coronavirus has created a global pandemic. Affected patients may develop acute lung injury and its more severe form - acute respiratory distress syndrome. Hypoxia and severe inflammation increase the production of vascular endothelial growth factor (VEGF), which induces vascular endothelial proliferation. Administration of the anti-VEGF monoclonal antibody bevacizumab is proposed for usage in moderate to severe pneumonia. We aim to present two cases of COVID-19 induced atypical pneumonia, which were treated with the anti-VEGF monoclonal antibody bevacizumab.

## Introduction

The severe acute respiratory syndrome (SARS) and the Middle East respiratory syndrome (MERS) began to emerge in 2002 and 2012. Recently emerged SARS-CoV-2 coronavirus causes atypical pneumonia. The first case was reported in Wuhan, China, in December 2019. The World Health Organization (WHO) declared the novel coronavirus (COVID-19) outbreak a global pandemic on March 11, 2020 [1]. Worldwide, more than 200 million cases with over 4.5 million deaths have occurred. In India, over 30 million cases with more than 0.4 million deaths, and still counting, have occurred [2-3]. 

COVID19 SARS CoV-2 virus is responsible for atypical pneumonia in afflicted people. The virus causes an inflammatory response, which facilitates viral replication, propagation, tissue damage, and hypoxia. Tissue hypoxia activates the hypoxia-induced factor-1 (HIF-1). HIF-1 is one of the regulators of oxygen homeostasis in cells. The HIF-1 induces transcription of many genes, which induce angiogenesis, including vascular endothelial growth factor (VEGF). HIF-1 also upregulates the expression of Angiotensin-converting enzyme 2 (ACE-2) receptor genes, which play a key role in COVID-19 pathogenesis. In tumor cells, due to high cell turnover, prominent cellular hypoxia leads to upregulation of the HIF-1 pathway and angiogenesis, making the HIF-1/VEGF pathway a key target for oncotherapy [4-5].

Bevacizumab is one of the popular anti-VEGF monoclonal antibodies, approved by the USFDA in 2004, that is widely used for colorectal carcinoma, non-small cell lung carcinoma, renal carcinoma, recurrent glioblastoma, cervical carcinoma, and ovarian carcinoma [6]. It is also used for wet age-related macular degeneration (AMD) to prevent neovascularization [7].

## Case presentation

Case 1

**Figure 1:**
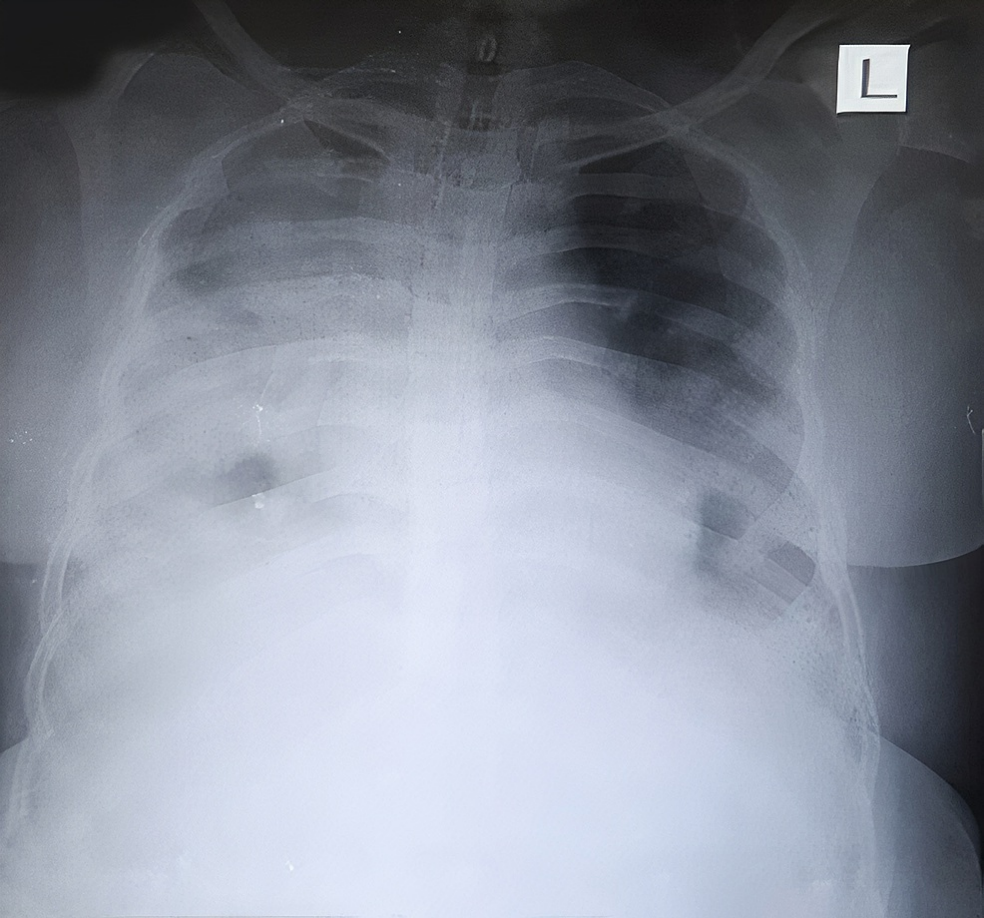
X-ray of case 1 before bevacizumab

**Figure 2:**
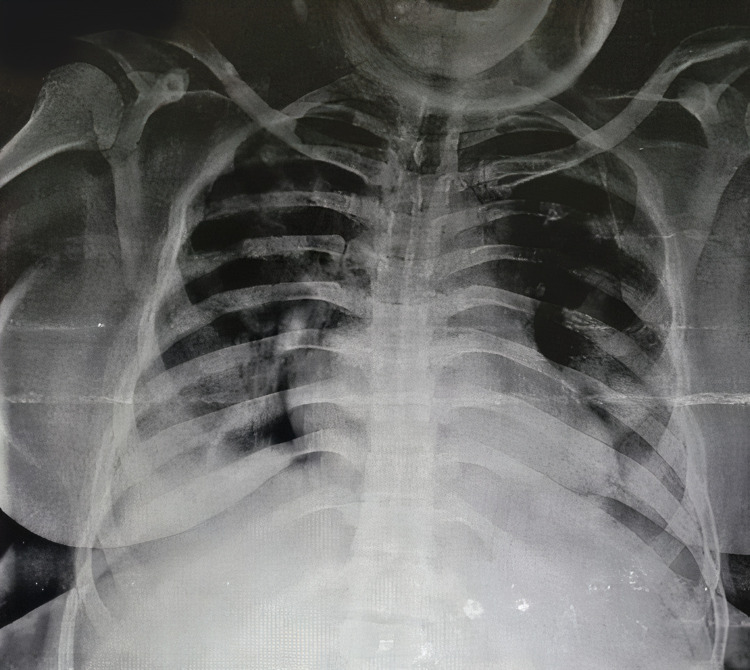
X-ray of case 1 after bevacizumab

**Figure 3:**
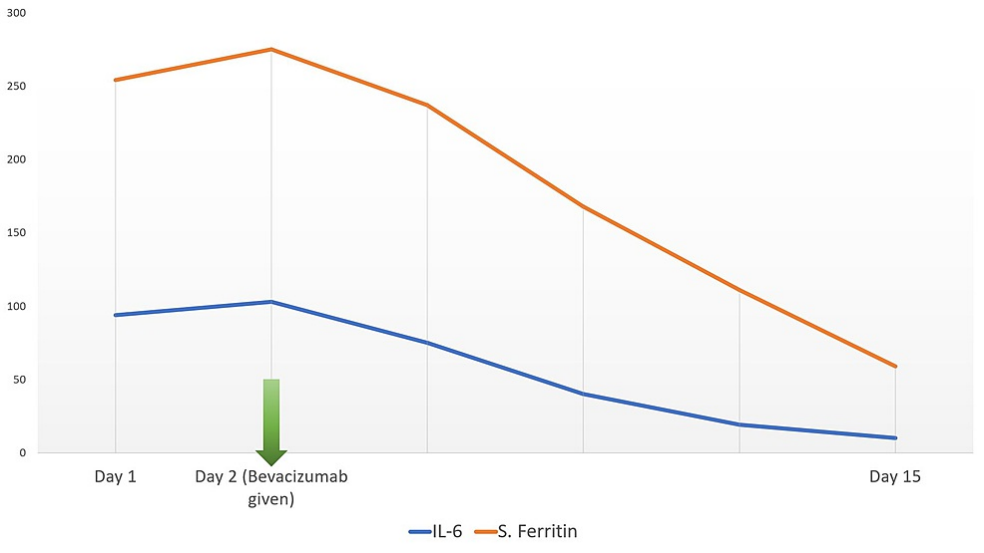
Inflammatory markers of case 1

A 33-year old female presented with fever, weakness, and dry cough for two days, and difficulty breathing for one day. COVID-19 reverse transcription polymerase chain reaction (RT PCR) test was positive with moderate viral load. The patient’s history was significant for diabetes mellitus and hypothyroidism. She was tachycardic and tachypneic on presentation to the ER. Pulse oximeter saturation with 12 L/min oxygen was 87%. After the admission, supplemental oxygen via a non-rebreather mask was started along with corticosteroids and remdesivir. The patient deteriorated and was shifted to noninvasive bi-level positive airway pressure (BiPAP) ventilation support. On day two, the patient's PaO2/FiO2 was 118. A standard dose of bevacizumab along with supportive treatment was administered. During consecutive days her clinical symptoms improved. On day seven, PaO2/FiO2 was 290. Moreover, the X-ray showed marked improvement in lung ground-glass opacity (Figure 1,2). On the same day, the patient was shifted to oxygen mask support. The patient’s laboratory investigation included white blood cell count, liver function, renal function, d-dimer, c-reactive protein (CRP), erythrocyte sedimentation rate (ESR), interleukin 6 (IL-6), and serum ferritin, which were within normal limits (Figure 3). 

Case 2

**Figure 4:**
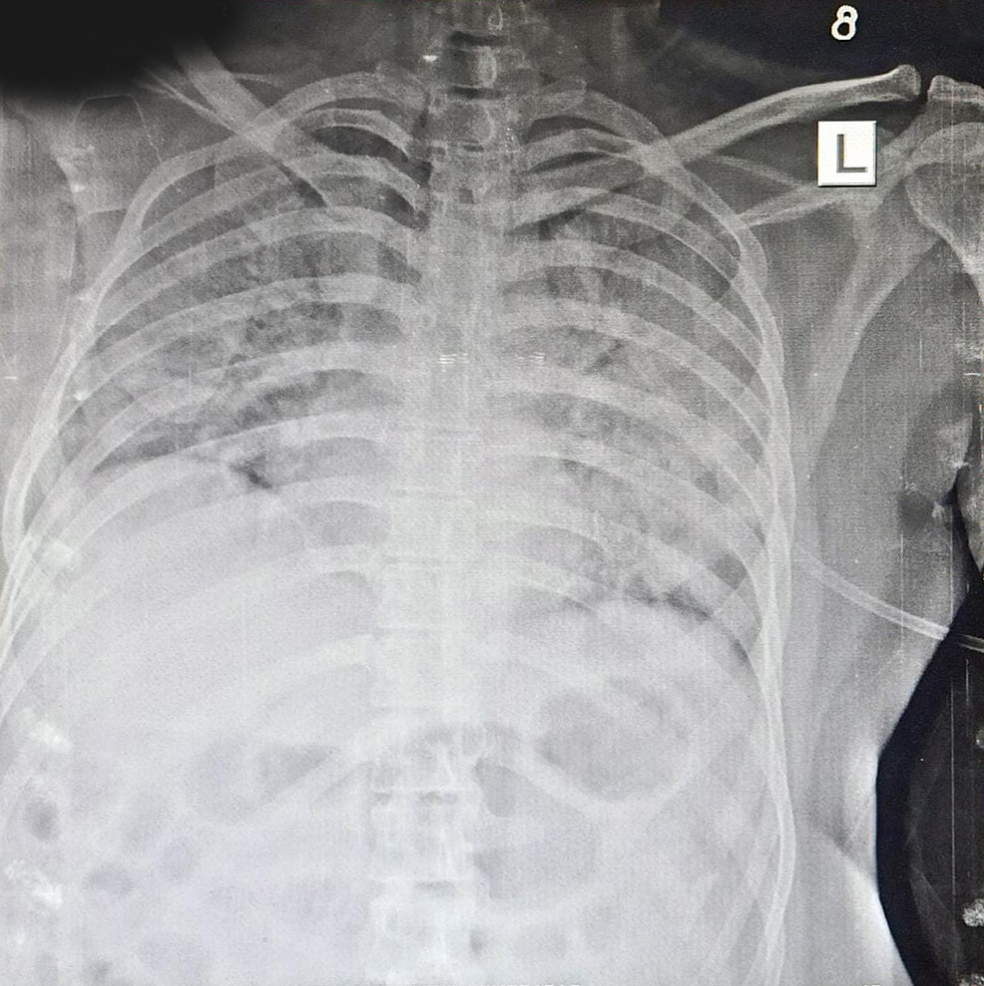
X-ray of case 2 before bevacizumab

**Figure 5:**
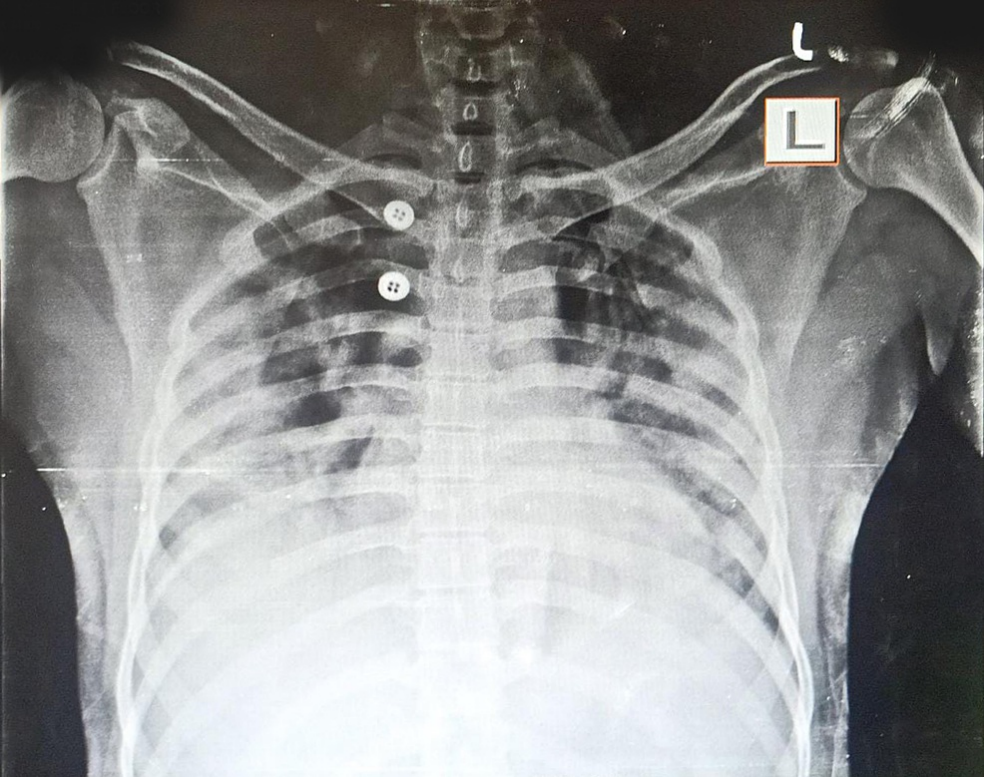
X-ray of case 2 after bevacizumab

**Figure 6:**
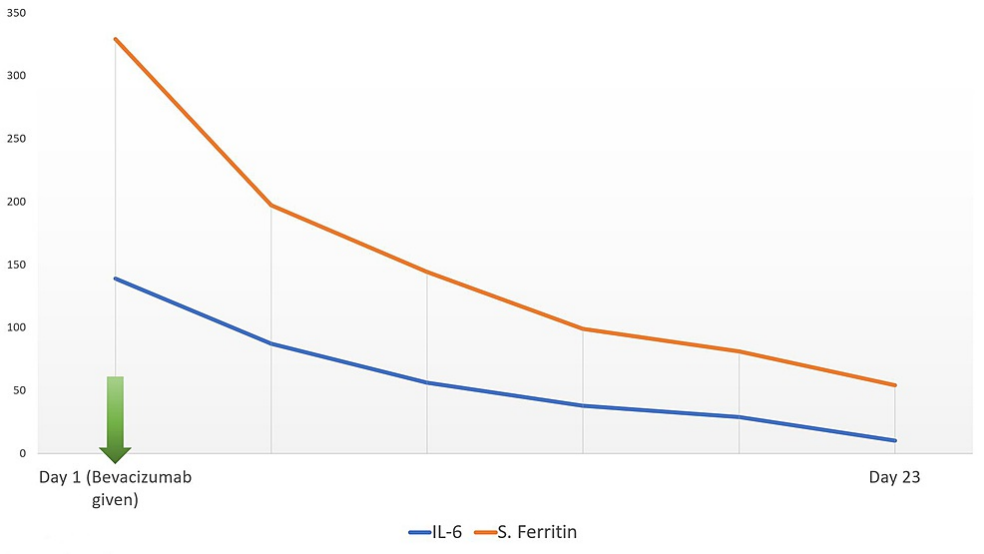
Inflammatory markers of case 2

A 30-year old male presented with difficulty breathing and fever for two days. COVID-19 RT PCR test came back positive with a high viral load. The patient was tachycardic and tachypneic, and his oxygen saturation reached 80% with oxygen support via a non-rebreather mask on presentation to ER. PaO2/FiO2 was 89, and the patient was shifted to noninvasive bi-level positive airway pressure (BiPAP) ventilation support. A standard dose of bevacizumab along with remdesivir, corticosteroids, and supportive treatment was given. On consecutive days his clinical symptoms improved. On day seven, chest X-ray showed improvement in ground-glass opacity (Figure 4,5), and PaO2/FiO2 increased to 240. On the 15th day since admission, he was shifted to an O2 mask. The patient’s laboratory investigation included white blood cell count, liver function, renal function, d-dimer, CRP, ESR, IL-6, serum ferritin, all of which were within normal limits (Figure 6). 

Outcome and follow-up

Patients started to improve clinically within 24 hours of Bevacizumab administration. Within seven days of admission, lung imaging of both patients showed improvement of ground-glass opacity, and PaO2/FiO2 ratio also improved. Patient 1 was discharged on day 15, and patient 2 was discharged on day 23 since admission. At that time, their COVID-19 PCR tests were negative.

Both of the patients recovered completely without any complications or noticeable bevacizumab side effects. Common adverse events include epistaxis, hemoptysis, gastrointestinal bleeding, hematemesis, intracerebral hemorrhage, vaginal bleeding, fatal hemorrhagic events. Subsequent outpatient clinic follow-up showed continued improvement of pulmonary function tests.

## Discussion

SARS-CoV-2 is transmitted via respiratory droplets and aerosols from person to person. Once inside the body, the virus binds to host receptors and enters cells through endocytosis or membrane fusion. ACE-2 has been identified as a functional receptor for SARS-CoV-2 and is highly expressed on the pulmonary epithelial cells and nasal epithelia. Next, the virus undergoes local replication and propagation in the cell. Newly formed viruses infect adjacent tissue. At this stage, viral particles are starting to migrate from the nasal epithelium to the upper and lower respiratory tract via the conducting airways. Infected pneumocytes release many different inflammatory markers and cytokines such as tumor necrosis factor-α (TNF-α), interleukins (IL-1, IL-6, IL-8, IL-120, and IL-12), interferon-λ and β, CXCL-10 and monocyte chemoattractant protein-1 (MCP-1). This cytokine storm acts as a chemo-attractant for neutrophils, CD4 helper T cells, and CD8 cytotoxic T cells, which begin to get sequestered in the lung tissue. These cells are responsible for fighting the virus. While this mechanism they cause subsequent inflammation and lung injury. The host cell undergoes apoptosis by releasing new viral particles, which then infect the adjacent type 2 pneumocytes. Continuous inflammation and viral replication cause persistent pneumocyte injury, leading to diffuse alveolar damage, eventually ending up in acute respiratory distress syndrome [8-12].

The incubation period for COVID-19 is 4-14 days from exposure to symptoms onset. The most common symptoms are fever, cough with or without expectoration, breathing difficulty, fatigue, headache from mild to severe condition. The clinical course is divided into mild, moderate, and severe conditions [13-14]. Patients can be treated in outpatient and inpatient services depending on the severity. Standard treatment includes antiviral Remdesivir, corticosteroids with or without oxygen support, and other supportive management. For severe conditions, rational use of baricitinib, tofacitinib, tocilizumab, and sarilumab is considered in newer guidelines [14-17].

**Figure 7:**
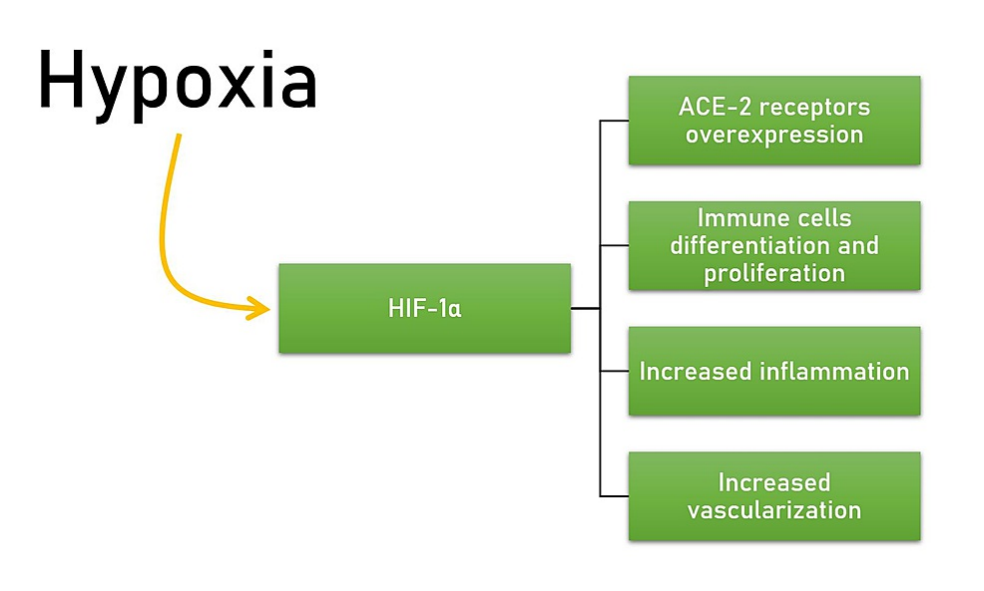
Effects of hypoxia HIF-1 - hypoxia-induced factor-1, ACE-2 - angiotensin converting enzyme 2

In COVID-19 patients, hypoxia induces the release of cytokines, inflammatory markers, and vascular endothelial growth factors, which cause the proliferation of endothelial cells of blood vessels and increase permeability, leading to pulmonary edema and a consequent decrease in alveolar ventilation (Figure 7). Recent evidence has revealed higher VEGF levels in COVID-19 patients than healthy controls [18-20].

## Conclusions

In the global pandemic of COVID-19, the need for effective treatment with minimum adverse events should be prioritized to decrease mortality. From February to April 2020, bevacizumab treatment was given to 26 patients in China and Italy, which showed significant improvement. These two cases also highlight the high efficacy of bevacizumab for SARS-CoV-2 patients. Significant clinical improvement of these patients led us to think about the need for a larger scale bevacizumab trial.
